# Analysis of human chorionic gonadotropin glycoforms by nano-liquid chromatography-mass spectrometry: novel strategies based on mobile phase additive and adduct dissociation

**DOI:** 10.1007/s00216-026-06620-0

**Published:** 2026-06-20

**Authors:** Pierre Besson-Magdelain, Christophe Chendo, Audrey Combès, Valérie Pichon, Nathalie Delaunay

**Affiliations:** 1 Laboratory of Analytical, Bioanalytical Sciences and Miniaturization, Chemistry, Biology and Innovation (CBI) UMR 8231, ESPCI Paris PSL, CNRS, PSL Research University, Paris, France; 2 Sorbonne Université, Paris, France; 3 UMR CBI 8231, ESPCI Paris PSL, 10 rue Vauquelin, 75231 Paris cedex 05, France

**Keywords:** Glycoform, Human chorionic gonadotropin, Nano-liquid chromatography, High-resolution mass spectrometry

## Abstract

The nano-LC-HRMS method previously developed for the analysis of human chorionic gonadotropin (hCG) glycoforms at the intact level involved trifluoroacetic acid (TFA) as ion-pairing agent in the LC mobile phase to favor separation, but resulted in both a significant decrease in the mass spectrometry (MS) signal and in complex mass spectra due to the presence of numerous adduct forms leading to a time-consuming manual data treatment and contributing to the MS signal decrease. To find the best balance between LC resolution, ESI ionization, and absence of adduct detection, two main strategies were here explored for the first time for hCG characterization. To enhance MS signal and reduce adduct formation, a decrease from 0.05 to 0.025% of TFA concentration was first assayed. It led to an efficient increase in MS signal intensities, but high glycoform/adduct ratios were still detected. The switch from TFA to difluoroacetic acid (DFA) 0.05% significantly improved the intensity of the signal in MS and significantly decreased adduct ratios (from 30 to 10%), despite some co-elution of hCG alpha (hCGα) and beta (hCGβ) glycoforms during LC separation. Keeping DFA as an ion-pairing agent, the All Ion Fragmentation mode of the Orbitrap mass spectrometer efficiently dissociated the remaining adducts leading to an increase in MS signal intensity by a factor 3 to 11 for hCGα glycoforms and 6 to 23 for hCGβ glycoforms. With these conditions, 76 hCGα and 200 hCGβ glycoforms were detected, resulting in a significant increase in the number of glycoforms detected compared with the initial method: a twofold increase for hCGα and a sixfold increase for hCGβ, thereby enabling a more detailed characterization of hCG glycosylation.

**Graphical abstract:**

**Figure:**
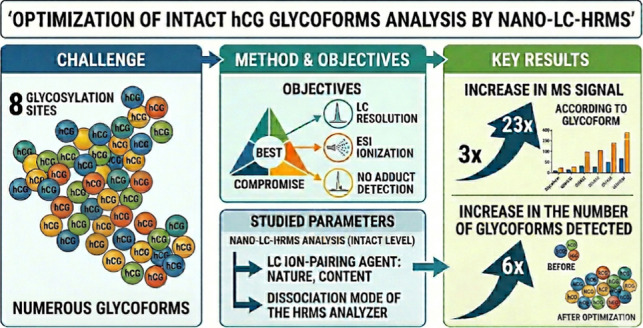

## Introduction

Human chorionic gonadotropin (hCG), better known as the pregnancy hormone, is composed of two non-covalently linked subunits, hCGα and hCGβ. hCGα contains two N-glycosylation sites on two asparagine residues, Asn-52 and Asn-78. hCGβ contains two N-glycosylation sites on Asn-13 and Asn-30 and four O-glycosylation sites on serine residues: Ser-121, Ser-127, Ser-132, and Ser-138 [1]. The presence of these eight glycosylation sites leads to numerous hCG glycoforms, and it was demonstrated that certain abnormal hCG glycosylation patterns can be associated with pregnancy-related illness. For example, trisomy 21 (Down syndrome) expresses a different glycosylation pathway compared to control [2, 3]. It was also shown that hyperglycosylated hCGβ has an impact on breast cancer growth and metastasis [4]. Therefore, there is a need to characterize hCG glycosylation.

Glycoproteins can be characterized using three main analytical strategies. The first one and the most commonly used is the bottom-up approach. It involves an enzymatic digestion followed by glycopeptide analysis using liquid chromatography (LC) coupled to mass spectrometry (MS) [5–7]. The second one is the glycan release approach involving an enzymatic deglycosylation step followed by derivatization of the glycans prior to their analysis by capillary electrophoresis (CE) or LC often coupled with MS [7, 8]. The third one consists in analyzing the intact glycoforms without any pre-treatment using CE or LC coupled to MS [9–11]. These three approaches are complementary and therefore all valuable. For example, only analysis at the level of intact molecules allows us to determine the structure and number of glycoforms.

Regarding hCG, several studies were carried out using at least one of the three analytical strategies mentioned above. The bottom-up approach was by far the most common one used for hCG [12–15], even if the one based on the released glycans was also used [15–17]. Regarding the analysis of hCG at the intact level to characterize its glycoforms, capillary zone electrophoresis with UV or MS detection [17, 18] and LC-MS with RP or HILIC modes have been used [14, 19–23]. Toll et al. were the first to analyze hCG by LC-MS and to observe two wide peaks using a trifluoroacetic acid (TFA)–based mobile phase, one for hCGα and one for hCGβ glycoforms [14]. Nevertheless, the lack of resolution of the used mass spectrometer (ion trap quadrupole) only allowed a tentative identification of the glycan present on a dozen glycoforms of each subunit. Our group proposed next a method involving reverse phase LC using FA as mobile phase additive coupled to high-resolution mass spectrometry (HRMS) using a TOF analyzer leading to the detection of about 30 hCGα glycoforms when analyzing Ovitrelle®, a recombinant hCG-based drug [20]. This method was subsequently implemented by Lebede et al., but this time using an Orbitrap as the analyzer and combining it with bottom-up and glycan analysis approaches [21]. Using also an Orbitrap, we switched from LC to nano-LC [22], to benefit from the nano-LC advantages [24]. Sensitivity was improved by a factor of 40, enabling not only the detection but also the identification of the 30 glycoforms of hCGα in Ovitrelle®. However, no hCGβ glycoforms were detected probably due to the larger number of glycans (6 for the beta subunit versus 2 for the alpha), which must interfere with the ionization step. Therefore, TFA was used as substitute of FA as ion-pairing agent to improve the separation, which effectively allowed the elution of the hCGα and hCGβ glycoforms into two resolved chromatographic massifs [23]. It resulted in an increase in the number of hCGα glycoforms detected from 30 to 42 analyzing Ovitrelle®. Most importantly, 33 hCGβ glycoforms were detected.

Unfortunately, it was observed that, while TFA allowed the separation of the hCGα and hCGβ glycoforms into two resolved chromatographic massifs, thereby improving the detection by HRMS of the hCGβ glycoforms, it also led to the formation of numerous adducts that a glycoform can form with one or several TFA molecules. This results in complex MS spectra that make data processing difficult and time-consuming, as it unfortunately has to be done manually. Moreover, numerous adducts spread the intensity of a given glycoform over several signals, lowering sensitivity. It was therefore necessary to improve again the nano-LC-HRMS method to further increase the number of glycoforms detected. To find the best balance between separation, ionization, and absence of adduct detection, two main strategies were explored. The first one aimed to reduce the mobile-phase ion-pairing content or to change its nature, replacing TFA by difluoroacetic acid (DFA). In the literature, its tendency to be a suitable alternative to TFA has been demonstrated for the analysis in LC-MS of small molecules [25] but also for larger biomolecules at the intact level (monoclonal antibodies, human erythropoietin (EPO), interferon-beta-1a (IFN-β−1a), antibody-drug conjugate, and growth factors) [26–29]. The second strategy investigated was to use a dissociation mode of the mass spectrometer to break the adducts. For this, two modes were evaluated, the All Ion Fragmentation (AIF) and the ion-source collision-induced dissociation (isCID). Additionally, a data processing with multiple criteria such as limit of intensity for glycoform MS signal and quality of the glycoform isotopic cluster was developed and used throughout this study to detect as many hCG glycoforms as possible with high confidence, reducing possible missing information.

## Experimental

### Chemicals and reagents

HPLC-grade acetonitrile (ACN) and LC-MS-grade water were supplied by Carlo Erba (Val de Reuil, France). Trifluoroacetic acid (TFA) and difluoroacetic acid (DFA) were supplied by Sigma-Aldrich (Saint-Quentin Fallavier, France). Ultra-pure water was obtained with a Milli-Q Purification System from Millipore (Molsheim, France). Ovitrelle® (Organon, Oss, The Netherlands) is a drug made of recombinant hCG from a Chinese hamster ovary cell line. Ovitrelle® contains 250 μg of hCG suspended in 500 µL of suspension liquid.

### Sample preparation

The hCG drug stock solution was desalted and filtered to remove additives and excipients from the formulation with ultra-centrifugal units (Microcon, Merck, Darmstadt, Germany) with a molecular weight (MW) cut-off of 10 kDa during 10 min, 4 °C at 10,000 rpm. After this step, the residues were washed with water and finally the samples were recovered from the inverted membrane by centrifugation for 2 min at 10,000 rpm and the volumes were adjusted with ultra-pure water to obtain a final concentration of 500 µg.mL^−1^ of hCG.

### Nano-LC-HRMS system

The analysis of hCG by nano-LC-HRMS was carried out using a nano-LC system (NC 3500 RS, Thermo Fisher Scientific, Courtaboeuf, France) coupled to an Orbitrap mass spectrometer (Exactive Plus, Thermo Fisher Scientific). The precolumn was a C18 PepMap (0.3 × 5 mm i.d., 300 Å) packed with 5-μm particles (Thermo Fisher Scientific) while the column was a Zorbax 300SB-C18 RSLC C18 (150 × 0.075 mm i.d., 300 Å) packed with 3.5-μm particles (Agilent Technologies, Les Ulis, France). The mobile phase was composed of (A) H_2_O/ACN (98/2; v/v) and (B) H_2_O/ACN (10/90; v/v) acidified with TFA at 0.05 or 0.025% v/v or DFA at 0.05 or 0.1% v/v or a mix of TFA and DFA at 0.025% each to obtain 0.05% v/v. The gradient starts at 10% of B, increasing the composition to 50% in 40 min, followed by a rapid increase to 90% in 5 min, a 5-min plateau, and a return to 10% in 5 min followed by 10 min of equilibration step. The column temperature was set at 60 °C. The injection volume and injection mode were 1 µL and partial loop mode, respectively. The flow rate was set at 300 nL.min^−1^. An emitter coupling the nano-LC system to the nanoESI source (EASY-spray source) of the mass spectrometer was added downstream the column. The EASY-spray source parameters were capillary voltage, 2.7 kV; capillary temperature, 320 °C; S-Lens, 60; sheath, auxiliary, and sweep gas flows at 0. The analyses were performed solely in positive ion mode, using full scan mode and, where indicated, AIF fragmentation with N₂ as the collision gas. The mass spectrometer was operated using a scan range from mass-to-charge (*m*/*z*) 1000 to 4000 with a scan rate of 1 spectrum/s. The mass resolution was set to 140,000 at *m*/*z* 200, the AGC target was 3e6, and the maximum injection time was 200 ms. For the isCID experiments, an EASY-spray C18 column (Acclaim PepMap300, 150 mm i.d. × 0.075 mm, 300 Å) packed with 5-µm particles (Thermo Fisher Scientific) was used. The same nano-LC parameters and conditions mentioned above were used. The EASY-spray source parameters were capillary voltage, 1.7 kV; capillary temperature, 280 °C; S-Lens, 60; sheath, auxiliary, and sweep gas flows at 0.

### Nano-LC-HRMS data processing

In this study, two manual data processing procedures were developed and used. The first one is associated to the optimization of the analytical method. Due to the complexity of the MS data and the presence of several hundreds of different glycoforms, several glycoforms were selected as reference to evaluate for each nano-LC-HRMS condition tested the impact on the adduct formation and the MS signal intensity. For each glycoform of reference, the isotopic profile was zoomed on the three most intense charge states. The intensities of the three most intense isotopes were collected and summed to obtain the overall MS intensity of the studied glycoform.

The second data treatment aims to determine the number of detected glycoforms and their elemental composition thanks to accurate mass measurements. Due to the intact-level analysis, only a general combination regrouping class of saccharides (hexose, hexose-N-acetylated, sialic acid…) was determined for a given glycoform without any precise saccharide identification (glucose, mannose, etc.…). For that, each massif on the chromatographic profile was divided into several sections of different time ranges, between 10 and 30 s, according to the TIC signal intensity of the considered time range. For example, close to the edge of the peak of interest where the signal intensity is low, the time range is large (i.e., 30 s) whereas when the signal intensity of the section of the chromatographic massif is high due to the co-elution of numerous glycoforms, the time range is reduced to 10 s to simplify as much as possible the resulting spectrum. For each section considered, the average mass spectrum was extracted and for each isotopic profile, the most intense peak was identified and the *m*/*z* value reported. This *m*/*z* value allowed the determination of the nature of the hCG subunit (alpha or beta) plus its potential saccharide composition.

## Results and discussion

### Limitations using the previously optimized nano-LC-HRMS method

**Fig. 1:**
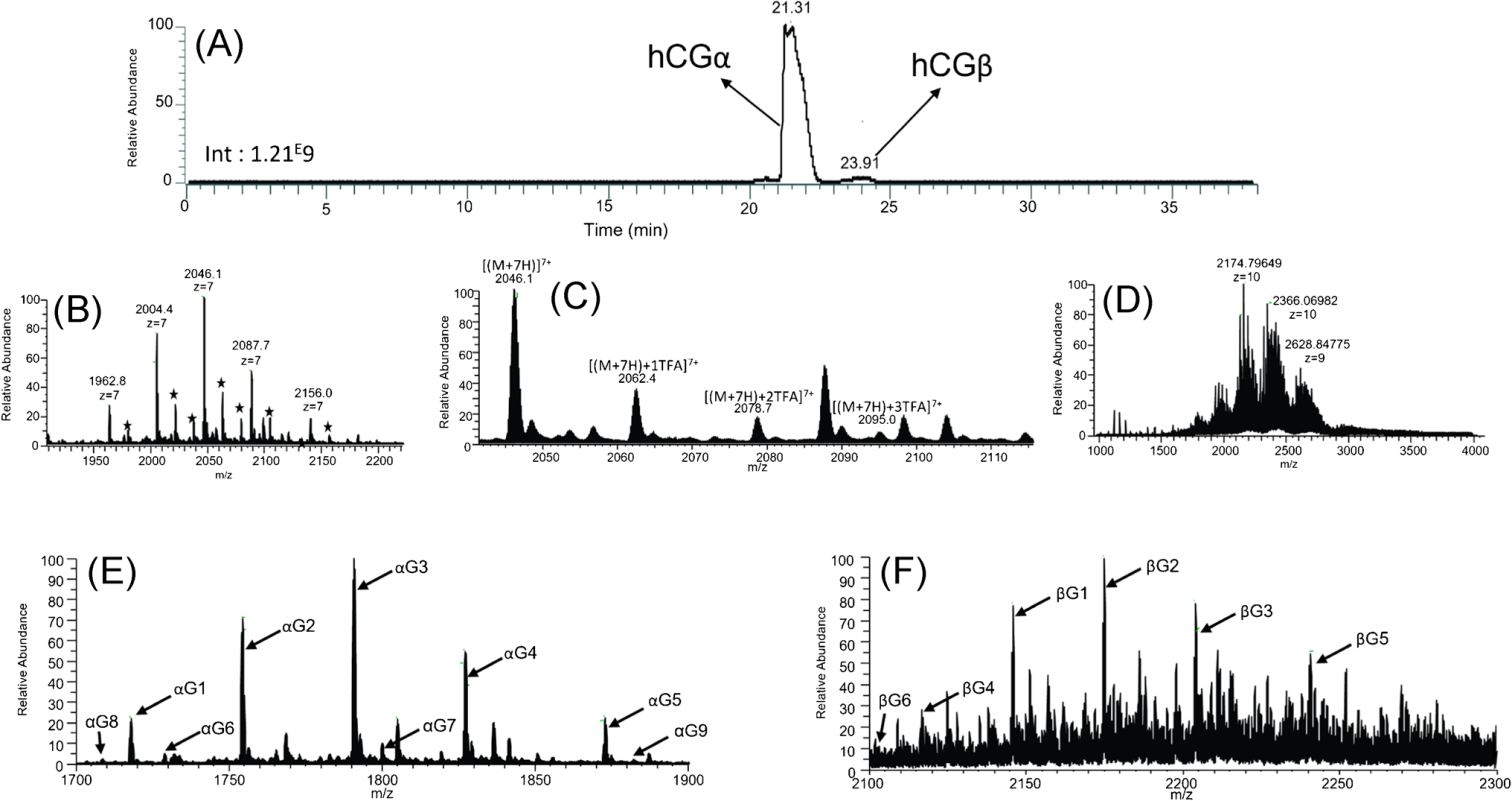
**A** BPC obtained by nano-LC-HRMS analysis of hCG at a concentration of 100 µg.mL^−1^ using TFA at 0.05%. **B** Average MS spectrum of the peak massif corresponding to hCGα glycoforms zoomed on charge state *z* =  + 7 with black stars corresponding to multiple adduct forms (*t*_R_, 20.0 min–22.7 min). **C** Zoom on the most intense glycoforms for *z* =  + 7 and the three adducts associated. **D** Average MS spectrum of the peak massif corresponding to hCGβ glycoforms (*t*_R_, 23.0 min–25.5 min). **E** MS spectrum of hCGα area zoomed on charge state *z* =  + 8 and the reference glycoforms (αG1–αG9). **F** MS spectrum of hCGβ area zoomed on charge state *z* =  + 10 and the reference glycoforms (βG1–βG6)

A nano-LC-HRMS method based on a C18 silica column and a mobile phase containing 0.05% of TFA as ion-pairing agent has been previously developed by our group [23]. The base peak chromatogram (BPC) obtained when analyzing hCG is reported in Fig. 1A. Two well-resolved massifs, one corresponding to hCGα glycoforms and the other to hCGβ glycoforms, appear. However, the resulting average mass spectrum provided in Fig. 1B for seven times charged hCGα glycoform ions illustrates the complexity of the resulting MS data. Indeed, many ions that could correspond to at least several dozen different glycoforms were detected such as glycoforms adducted to TFA, with the number of TFA adducted to one glycoform varying from 1 to 3 (Fig. 1C). Regarding the hCGβ glycoforms, the average mass spectrum presented in Fig. 1D highlights the drastically greater complexity of the MS signal obtained. To the best of our knowledge, there is no specialized software for identifying glycoforms. Therefore, the use of an automated data processing could lead to a very large overestimation of the number of glycoforms or wrong attributions. This is why a manual data processing was done.

Thus, to save time, we proposed in this study to arbitrarily select several hCGα glycoforms as reference glycoforms: four glycoforms so-called αG1 through αG4 giving among the most intense MS signals, as well as three medium-intense glycoforms (αG5 to αG7) and two low-intense glycoforms (αG8 and αG9). Similarly, three intense hCGβ glycoforms (βG1 through βG3), one medium-intense hCGβ glycoform (βG4), and two low-intense hCGβ glycoforms (βG5 and βG6) were selected. Figure 1E and F presents the selected glycoforms and illustrates the need to select multiple reference glycoforms regarding the complexity of the MS data. It can be seen that their intensities can differ by more than two orders of magnitude. In addition, their theoretical monoisotopic masses are given in the first column of Table 1.

**Table 1:** Theoretical maximum isotopic mass of hCGα and hCGβ glycoforms used as reference plus their retention times and their EIC peak intensity with their RSD values analyzing hCG by nano-LC-HRMS with 0.05% DFA (*n* = 3). Other nano-LC-HRMS conditions: see section “Nano-LC-HRMS system”

Glycoform of reference	Theoretical maximum isotopic mass	Retention time (min)	EIC peak intensity (RSD)
αG1	13,732.86585	19.56 ± 0.04	1.65E+7 (2%)
αG2	140,23.96143	19.68 ± 0.02	2.50E+7 (3%)
αG3	14,316.05886	20.06 ± 0.07	2.02E+7 (4%)
αG4	14,607.15445	20.37 ± 0.01	1.49E+7 (3%)
αG5	14,972.28687	20.24 ± 0.00	3.98E+6 (3%)
αG6	13,820.88195	19.77 ± 0.04	2.54E+6 (11%)
αG7	14,389.09569	19.61 ± 0.05	3.96E+6 (1%)
αG8	13,658.82902	19.81 ± 0.02	1.28E+6 (7%)
αG9	15,046.32370	19.70 ± 0.04	6.46E+5 (17%)
βG1	21,446.84234	21.49 ± 0.02	6.16E+5 (13%)
βG2	21,735.88458	21.69 ± 0.09	5.62E+5 (6%)
βG3	21,154.60470	21.82 ± 0.11	4.70E+5 (5%)
βG4	22,029.03345	21.36 ± 0.04	4.08E+5 (8%)
βG5	22,393.02373	20.97 ± 0.02	3.58E+5 (5%)
βG6	20,497.37413	21.19 ± 0.03	1.31E+5 (6%)

**Fig. 2:**
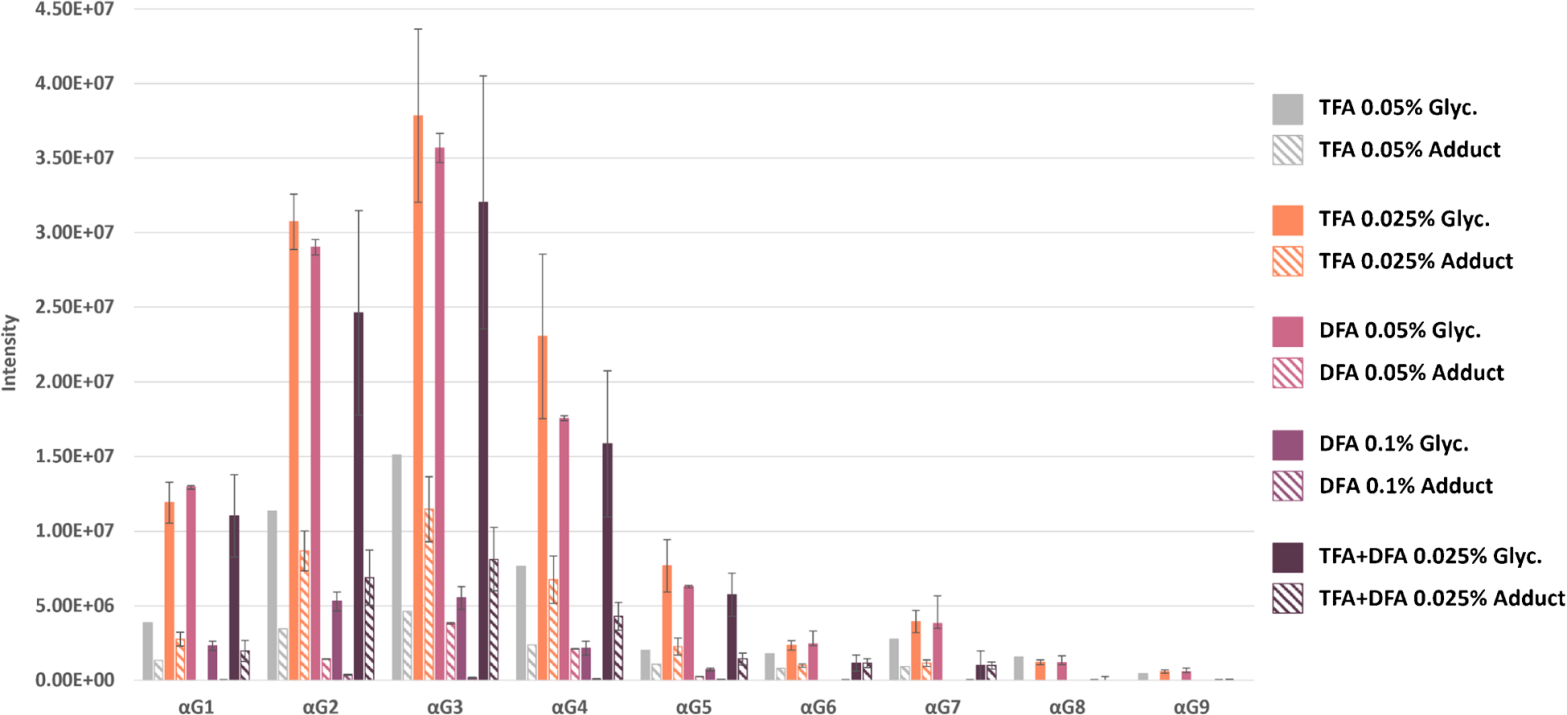
Sum of the intensities of the 3 most intense MS peaks at 3 different charge states (*z* =  6 to + 8 for TFA and *z* =  7 to + 9 for DFA) for the hCGα reference glycoforms and their corresponding adduct forms using TFA at 0.05%, TFA at 0.025%, DFA at 0.05%, DFA at 0.1%, and TFA/DFA mixture, each at 0.025% (*n* = 3). Nano-LC-HRMS conditions: see section “Nano-LC-HRMS system”. Data processing method: see section “Nano-LC-HRMS data processing”

**Fig. 3:**
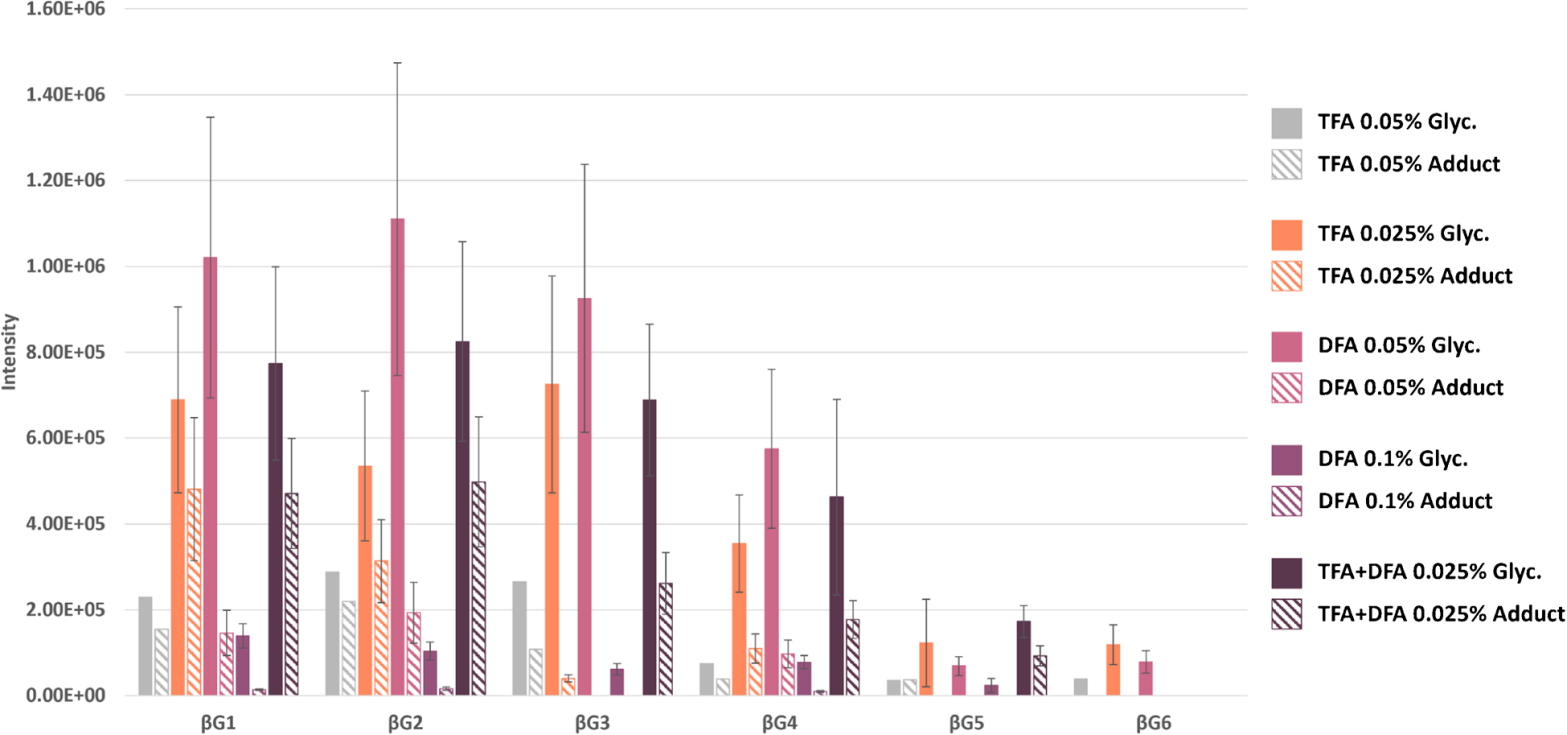
Sum of the intensities of the 3 most intense MS peaks at 3 different charge states (*z* =  +6 to +8 for TFA and *z* =  +7 to +9 for DFA) for the hCGβ reference glycoforms and their corresponding adduct forms using TFA at 0.05%, TFA at 0.025%, DFA at 0.05%, DFA at 0.1%, and TFA/DFA mixture, each at 0.025% (*n* = 3). Nano-LC-HRMS conditions: see section “Nano-LC-HRMS system”. Data processing method: see section “Nano-LC-HRMS data processing”

Figures 2 and 3 present the sum of the intensities of the three most intense MS peaks at three different charge states, z =  + 6 to + 8 for TFA at 0.05% for hCGα and hCGβ reference glycoforms, respectively. The ratio between the MS intensity of each glycoform and its adduct forms (sum of the adducts with one, two, and three molecules of TFA) was calculated for the intense and medium-intense glycoforms with these initial analytical conditions using 0.05% of TFA (the intensities of the adduct forms were too low to detect them for the low-intense hCGα and hCGβ glycoforms of reference). The proportion of adduct forms was between 17 and 44% for the hCGα glycoforms and 41 and 100% for the hCGβ. These high values have led us to review the analysis conditions in order to find a better compromise between chromatographic separation of glycoforms, electrospray ionization, and reduction of the proportion of adduct forms to enhance the MS signal of glycoforms while simplifying the manual processing of MS data. This would also allow the detection of additional glycoforms with low signal and/or low concentration.

### Modification of the ion-pairing agent content and nature

Two different approaches were first considered: the diminution of the TFA content, which had not been tested in our previous study [23], or the use of DFA as ion-pairing agent. Indeed, it was reported in the literature that DFA can be an alternative to TFA for other biomolecule analysis to improve the electrospray ionization efficiency and MS sensitivity [26–29]. This is why it seemed interesting to investigate the potential of DFA for the analysis of hCG glycoforms. In both approaches, the main objective was to maintain good chromatographic resolution while maximizing the ionization of hCG glycoforms and minimizing the adduct form detection.

**Fig. 4:**
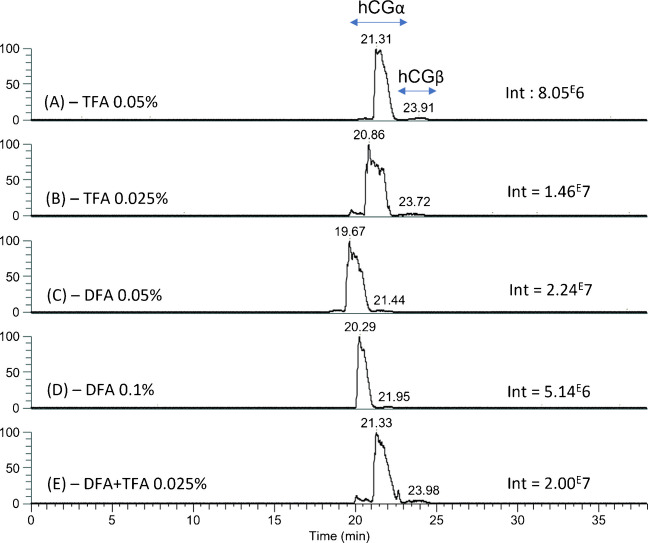
BPCs obtained by nano-LC-HRMS analysis of hCG at a concentration of 100 µg.mL^−1^ using **A** TFA at 0.05%, **B** TFA at 0.025%, **C** DFA at 0.05%, **D** DFA at 0.1%, and **E** TFA/DFA mixture, each at 0.025%. For other nano-LC-HRMS conditions, see section “Nano-LC-HRMS system”

The first approach was to reduce the TFA content in the nano-LC mobile phase, from 0.05 to 0.025%. As expected, a decrease in the chromatographic resolution between the two massifs was observed but they remain separated at the baseline (Fig. 4A and B). Regarding the MS signal that is reported in Figs. 2 and 3 for hCGα and hCGβ reference glycoforms respectively, a gain of a factor of 2 to 5 was observed. It confirmed that, as expected, reducing ion-pairing agent content decreased the ion-suppression effect and thereby maximized the ionization of the hCG glycoforms. Nevertheless, the glycoform/adduct form ratios are still above 30% for all the hCG glycoforms of reference. This indicates that reducing the ion-pairing agent content did not reduce, however, the formation of TFA-adducts. The complexity of the mass spectrum and the ion-suppression effect induced by the adduct form detection is still the major issue of the analytical method. This is why another strategy was considered.

The performance of DFA as ion-pairing agent was then investigated. To this end, DFA was introduced at the same concentration as TFA, i.e., 0.05%. The resulting base peak chromatogram presented in Fig. 4C shows that retention and resolution were significantly reduced compared to those obtained with TFA, with two massifs partially co-eluted and lower retention times (21.4 and 24.0 min with TFA versus 19.8 and 21.5 min with DFA). This effect on retention time had already been reported and discussed [29]. In terms of resolution, DFA has a lower acidity than TFA and is less likely to form ion pairs, making it less effective than TFA at minimizing secondary interactions with the stationary phase. Regarding the MS peak intensities reported in Figs. 2 and 3, DFA led to an increase in intensity by a factor of at least 2 and up to 5 for all the hCG glycoforms of reference, except for the hCGα glycoforms of low intensity compared to MS signal obtained with TFA at the same concentration. Moreover, the glycoform/adduct form ratios were reduced by a factor of at least 2. Additionally, some adduct forms of glycoforms were no longer detected with DFA at 0.05%, even for intense or medium-intense glycoforms such as for αG1, αG6, αG7, and βG3. The adducts may still be formed, but at a level below the detection limit of the analytical method.

As DFA at 0.05% induced a co-elution of the two chromatographic massifs, the content of DFA was increased to 0.1%. As it can be observed in Fig. 4D, the resolution between the two chromatographic massifs was improved, eliminating the co-elution. This enhancement may reduce the ion-suppression effect between co-eluted hCG glycoforms and thus increase the MS sensitivity. Nevertheless, Figs. 2 and 3 show a drastic decrease in MS peak intensity. For medium- and low-intensity glycoforms of reference, some are even no longer detected. In conclusion, whether using TFA or DFA, the same behavior was observed: a lower content of ion-pairing agent results in lower chromatographic resolution but higher MS peak intensity.

**Fig. 5:**
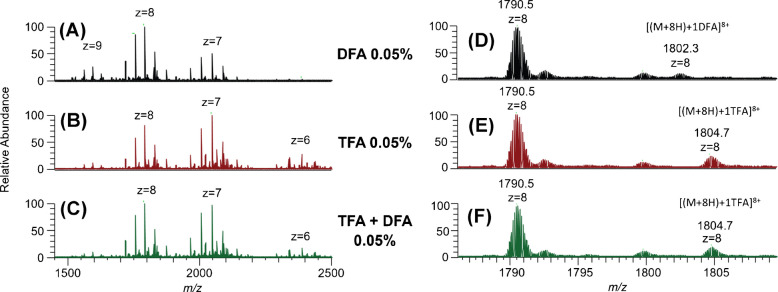
Average mass spectra of the chromatographic peak massif corresponding to the hCGα glycoforms using **A** DFA at 0.05% (*t*_R_, 19.3–21 min), **B** TFA at 0.05% (*t*_R_, 20.0–22.7 min), or **C** a DFA/TFA mixture, each at 0.025% (*t*_R_, 20.9–22.7 min). Zoom on the most intense glycoform ions for *z* =  + 8 and the first adduct associated with one molecule of ion-pairing agent without dissociation method: **D** DFA at 0.05%, **E** TFA at 0.05%, or **F** TFA/DFA mixture, each at 0.025%. Nano-LC-HRMS conditions: see section “Nano-LC-HRMS system”

The major issue when using DFA is the partial co-elution of the two chromatographic massifs, whereas it was not the case with TFA. Therefore, the use of a mixture of DFA and TFA at 0.025% each (i.e., a total of 0.05%) was tested to obtain a better compromise between LC resolution and MS sensitivity. The resulting chromatogram is presented Fig. 4E. Regarding the MS signals presented in Figs. 2 and 3, for most of the reference glycoforms the intensities are quite similar using TFA 0.025% or the mixture DFA/TFA. Figure 5 presents the full average mass spectra corresponding to the first chromatographic peak massif using 0.05% of DFA (Fig. 5A), 0.05% of TFA (Fig. 5B), and DFA/TFA mixture, each at 0.025% (Fig. 5C). The same charge state distribution is observed for the TFA and TFA/DFA mixture (i.e., the three most intense charge distribution *z* =  + 6, *z* =  + 7, and *z* =  + 8) whereas it is different for DFA with the tested conditions (i.e., the three most intense charge distribution *z* =  + 7, *z* =  + 8, and *z* =  + 9). A zoom of the MS spectra at charge state *z* =  + 8 corresponding to one glycoform and its first adduct form associated is presented using DFA 0.05% (Fig. 5D), TFA 0.05% (Fig. 5E), and DFA/TFA mixture each at 0.025% (Fig. 5F). It can be clearly observed that the DFA adduct form (*m*/*z* 1802.3) is not detected using the DFA/TFA mixture whereas the TFA adduct form (*m*/*z* 1804.7) is clearly present. It should be noted that this phenomenon is the same whatever the glycoform and its adduct forms (data not shown). All those results reflect the fact that hCG glycoforms have more affinity for TFA than for DFA. Therefore, it seems a better compromise to select DFA alone as ion-pairing agent to limit the formation of adducted forms, even if some co-elution of the hCGα and hCGβ glycoforms occurs, which can disturb their ionization in electrospray. Thus, a percentage of 0.05% of DFA in mobile phase was retained as the best compromise for the characterization of hCG glycoforms. Table 1 presents the retention time and the peak intensity of each hCG glycoform of reference with this LC condition. It can be observed that good repeatability was obtained for both parameters, with retention time RSD values below 1% and peak intensity RSD values below 17% with an average close to 6% (*n* = 3).

### Potential of MS modes for adduct dissociation

The second method for limiting the impact of adducts, which cannot be avoided in the presence of additives necessary for the separation of glycoforms, is to attempt to dissociate them. For this, two dissociation techniques inherent to the Orbitrap mass spectrometer used were evaluated. However, it is important to bear in mind that high energy input could fragment the glycoforms along with the adducts, resulting in a loss of information. For both dissociation techniques, initial tests were performed using 0.05% TFA in the mobile phase to compare with the initial analytical conditions and confirm the affinity difference between TFA and DFA for glycoforms.

**Fig. 6:**
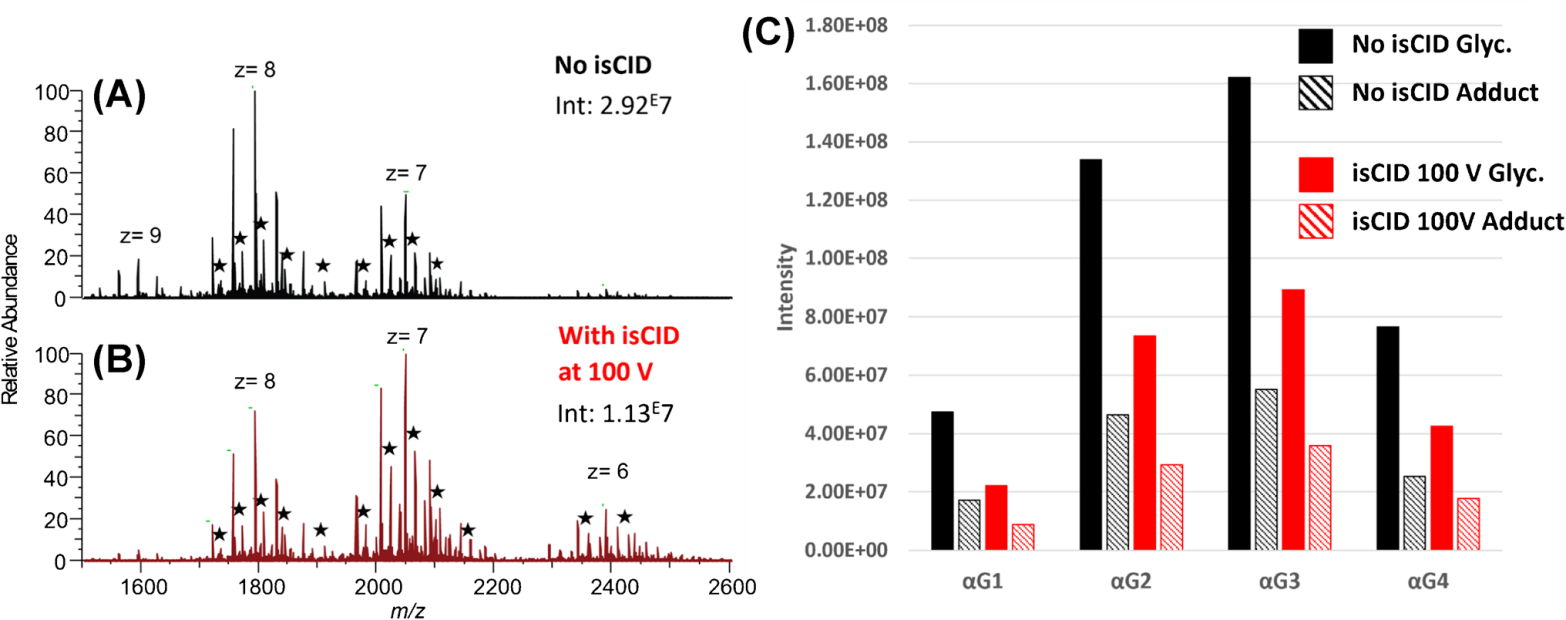
Average mass spectra of the chromatographic peak massif corresponding to the hCGα glycoforms using TFA at 0.05% **A** without dissociation method (*t*_R_, 20.9–24.1 min) or **B** coupled with isCID at 100 V (*t*_R_, 21.2–24.3 min). **C** Sum of the intensities of the 3 most intense peaks on 3 charge states of the 4 intense hCGα glycoforms of reference. Black stars, adduct forms. Nano-LC-HRMS instrumental method: see section  “Nano-LC-HRMS system”. Data processing method: see section “Nano-LC-HRMS data processing”

The first dissociation technique evaluated was the in-source collision-induced dissociation (isCID). In this mode, an additional voltage is applied in the ionization source to accelerate the ions freshly formed to collide with the air at atmospheric pressure. Since the collision should cause ion dissociation, it was expected that it would dissociate the adduct forms before their detection, as it was the case for the dissociation adduct forms of intact glycoproteins (IFN-β−1a and EPO) [26]. The isCID voltage is limited to 100 V for the instrument used in this study, so this is the voltage value selected. It is worthwhile to notice that these isCID experiments were the only ones carried out with an EASY-spray source (just before the supplier stopped selling the Easyspray columns) and quite different voltage parameters, which may explain the difference in the charge distribution profiles between Fig. 6A and B. Average mass spectra of the peak massif corresponding to the hCGα glycoforms obtained with 0.05% TFA without and with isCID dissociation are presented in Fig. 6A and B, respectively. Figure 6C presents the comparison of the MS signals of the intense hCGα reference glycoforms with and without isCID at 100 V. The glycoform/adduct form ratios were around 30% in both cases. Therefore, the isCID mode was not efficient in our case to dissociate TFA from glycoforms and was no longer evaluated in this study.

**Fig. 7:**
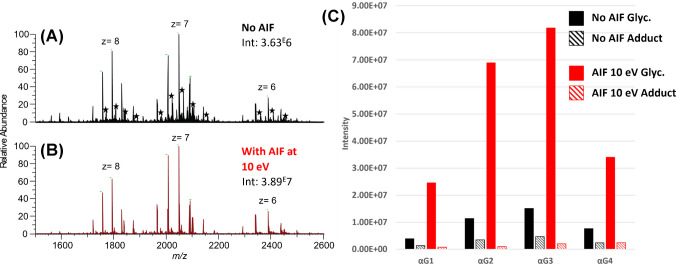
Average mass spectra of the chromatographic peak massif corresponding to the hCGα glycoforms using TFA at 0.05% **A** without dissociation method (*t*_R_, 20.0–22.7 min) or **B** coupled with AIF at 10 eV (*t*_R_, 20.5–23.2 min). **C** Sum of the intensities of the 3 most intense peaks on 3 charge states of the 4 intense hCGα glycoforms of reference. Black stars, adduct forms. Nano-LC-HRMS conditions: see section “Nano-LC-HRMS system”. Data processing method: see section “Nano-LC-HRMS data processing”

The second dissociation method tested was the All Ion Fragmentation (AIF) mode, where all ions within the full *m/z* range are introduced into the collision cell of the Orbitrap and submitted to collision-induced dissociation. The resulting average mass spectra of the peak massif corresponding to the hCGα glycoforms with or without using AIF are presented on Fig. 7A and B, respectively. First, a slight change between Figs. 6A and 7A can be observed, which can be explained by the use of the Easyspray source and column for isCID experiments as previously mentioned. Nevertheless, Fig. 7A and B can be directly compared, as they were obtained with the same analytical conditions, with the exception of the AIF implementation. The lowest energy value possible for the instrument (i.e., 10 eV) was applied. It can be observed that there is no modification of the charge state distribution, which may indicate that glycoform dissociation is not observed in addition with a less complex MS spectrum. The comparison of MS signals with or without AIF, shown in the histogram (Fig. 7C), highlights an impressive six-fold increase in the intensity of the MS signal for glycoforms. Furthermore, it should be noted that the ratio of adduct forms detected decreased from 30 without AIF to approximately 3% with AIF. Thus, the use of the AIF mode at 10 eV allowed the dissociation of numerous adduct forms and, as a result, their elimination from the mass spectra. This MS mode was thus used for the remainder of the study.

**Fig. 8:**
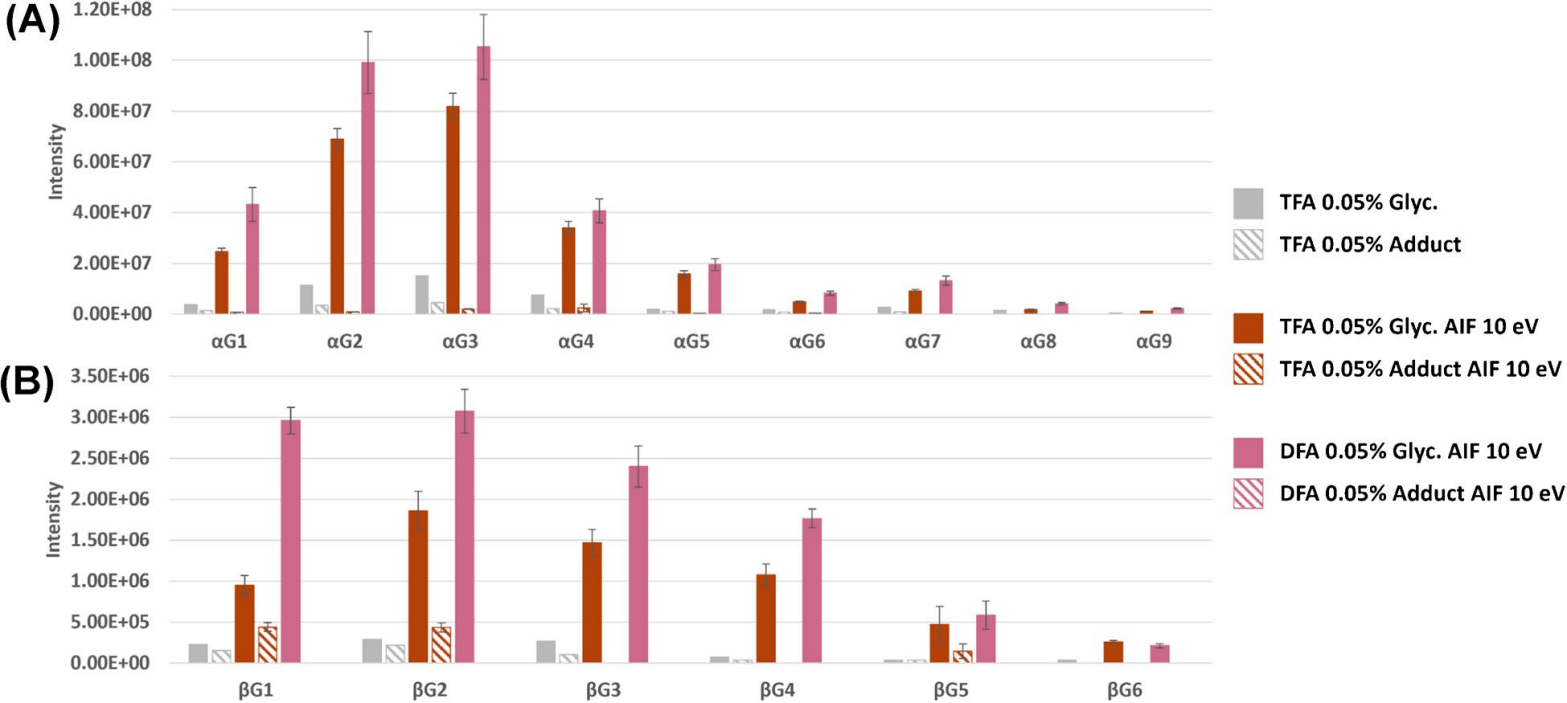
Sum of the intensities of the 3 most intense MS peaks on 3 different charge states of reference **A** hCGα and **B** hCGβ glycoforms using TFA at 0.05%, TFA at 0.05% plus AIF mode at 10 eV, and DFA at 0.05% plus AIF at 10 eV (*n* = 3). Nano-LC-HRMS conditions: see section “Nano-LC-HRMS system”. Data processing method: see section “Nano-LC-HRMS data processing”

Unfortunately, using TFA at 0.05% with AIF at 10 eV, some adduct forms were still detected by the analyzer. Therefore, the combination of the AIF mode with the use of DFA at 0.05% was studied. Figure 8A and B compares the MS intensities of the reference glycoforms and their adduct forms obtained with TFA or DFA at 0.05% both coupled with AIF mode at 10 eV and also TFA at 0.05% without AIF provided as reference conditions of this study. Using AIF and moving from TFA to DFA led to mainly the absence of adduct form detection. In comparison with the initial conditions, the MS signal was increased with a factor 3 to 11 for hCGα glycoforms and 6 to 23 for hCGβ glycoforms. The dissociation of adduct form represents a major advance in the data treatment process, not only in terms of time saved, but also in terms of peak intensity, which may enable the detection of a greater number of glycoforms, particularly those of low abundance.

### Evaluation of the gain in the number of glycoforms detected

To summarize, the use of DFA at 0.05% coupled with 10 eV AIF mode prevents the formation and detection of adduct forms and thereby increases the MS signal intensities for the glycoforms of interest, which appears to be a major advance. However, the chromatographic separation of the hCG glycoforms was slightly degraded by the replacement of TFA by DFA, which can make ionization of some glycoforms more difficult. To be sure that these new analytical conditions give better results than the ones we previously published [23], the total number of detected glycoforms was determined by applying both methods. To achieve this, manual data processing was carefully performed. Multiple criteria were implemented for this. First of all, a minimum intensity threshold was set for the isotopic patterns, as any pattern with a maximum intensity lower than 10^4^ was not considered. Additionally, several patterns overlapped (i.e., two isotopic patterns with overlapping peaks that can introduce interference and possibly incorrect information on the maximum isotopic peak) and were not considered for attribution. Lastly, any poorly defined isotopic pattern was excluded during this data processing. It is important to note that these parameters are drastic and may induce some loss of information, but this approach, despite being time-consuming, ensures that no incorrect data is interpreted (noise, MS artifact, etc.). Using 0.05% of TFA and analyzing the same recombinant hCG-based drug, the number of glycoforms detected was 42 for hCGα and 33 for hCGβ [23]. Using 0.05% DFA and the use of the 10 eV AIF mode, 76 hCGα and 200 hCGβ glycoforms were detected. All the detected glycoforms are detailed in Tables 2 and 3 (note that a glycoform was considered when it was detected on 3 successive independent nano-LC-HRMS analyses). This constitutes a strong improvement in the hCG glycoform characterization.

**Table 2:** Compilation of all the identified hCGα glycoforms in the hCG sample analyzed by nano-LC-HRMS using DFA at 0.05% and the AIF mode (10 eV): elemental composition, proposed monosaccharide composition, theoretical maximum isotopic mass, theoretical monoisotopic mass, and the error in ppm

Elemental composition	Proposed monosaccharide composition	Theoretical maximum isotopic mass	Theoretical monoisotopic mass	Mean error (ppm)
C_499_H_774_N_126_O_179_S_13_	HexNAc(4)Hex(5)NeuAc(0)	11,818.18590	11,811.17054	+ 1.4
C_510_H_791_N_127_O_187_S_13_	HexNAc(4)Hex(5)NeuAc(1)	12,109.28150	12,102.26595	+ 0.3
C_521_H_808_N_128_O_195_S_13_	HexNAc(4)Hex(5)NeuAc(2)	12,401.37879	12,393.36167	+ 0.3
C_553_H_863_N_129_O_219_S_13_	HexNAc(7)Hex(10)NeuAc(0)	13,238.69077	13,230.67277	+ 0.4
C_553_H_864_N_126_O_224_S_13_	HexNAc(4)Hex(14)NeuAc(0)	13,277.66401	13,269.64595	+ 0.8
C_556_H_867_N_129_O_222_S_13_	HexNAc(6)Hex(10)NeuAc(1)	13,326.70687	13,318.68882	0.0
C_558_H_870_N_130_O_222_S_13_	HexNAc(7)Hex(9)NeuAc(1)	13,367.73344	13,359.71536	− 0.7
C_560_H_873_N_131_O_222_S_13_	HexNAc(8)Hex(8)NeuAc(1)	13,408.76000	13,400.74191	− 0.5
C_561_H_876_N_130_O_224_S_13_	HexNAc(8)Hex(10)NeuAc(0)	13,441.77027	13,433.75214	− 0.3
C_562_H_877_N_129_O_227_S_13_	HexNAc(6)Hex(11)NeuAc(1)	13,488.75980	13,480.74164	0.0
C_564_H_880_N_130_O_227_S_13_	HexNAc(7)Hex(10)NeuAc(1)	13,529.78636	13,521.76819	0.0
C_564_H_878_N_130_O_228_S_13_	HexNAc(5)Hex(9)NeuAc(3)	13,543.76741	13,535.74745	0.4
C_567_H_885_N_133_O_224_S_13_	HexNAc(11)Hex(7)NeuAc(0)	13,564.84996	13,556.83179	− 5.9
C_564_H_881_N_127_O_232_S_13_	HexNAc(4)Hex(14)NeuAc(1)	13,566.75774	13,560.74136	− 2.5
C_566_H_883_N_131_O_227_S_13_	HexNAc(8)Hex(9)NeuAc(1)	13,750.81293	13,562.79474	+ 0.3
C_566_H_881_N_131_O_228_S_13_	HexNAc(6)Hex(8)NeuAc(3)	13,584.79219	13,576.7740	+ 0.3
C_567_H_884_N_130_O_230_S_13_	HexNAc(6)Hex(10)NeuAc(2)	13,617.80246	13,609.78423	− 0.2
C_569_H_887_N_131_O_230_S_13_	HexNAc(7)Hex(9)NeuAc(2)	13,658.82902	13,650.81078	+ 0.1
C_570_H_894_N_133_O_228_S_13_	HexNAc(8)Hex(6)NeuAc(3)	13,666.84532	13,658.82710	− 2.3
C_570_H_890_N_130_O_232_S_13_	HexNAc(7)Hex(11)NeuAc(1)	13,691.83930	13,683.82101	+ 0.5
C_572_H_893_N_131_O_232_S_13_	HexNAc(8)Hex(10)NeuAc(1)	13,732.86585	13,724.84756	− 0.1
C_574_H_896_N_132_O_232_S_13_	HexNAc(9)Hex(9)NeuAc(1)	13,773.89242	13,765.87412	− 3.6
C_574_H_901_N_132_O_233_S_13_	HexNAc(7)Hex(8)NeuAc(3)	13,787.87168	13,779.85337	− 2.3
C_576_H_900_N_130_O_234_S_13_	HexNAc(7)Hex(9)NeuAc(1)Fuc(3)	13,805.90746	13,797.88909	− 0.3
C_575_H_899_N_131_O_234_S_13_	HexNAc(9)Hex(11)NeuAc(0)	13,806.90268	13,798.88434	− 1.9
C_575_H_897_N_131_O_235_S_13_	HexNAc(7)Hex(10)NeuAc(2)	13,820.88195	13,812.86360	+ 0.4
C_576_H_900_N_130_O_237_S_13_	HexNAc(7)Hex(12)NeuAc(1)	13,853.89221	13,845.87383	− 2.1
C_575_H_898_N_128_O_240_S_13_	HexNAc(4)Hex(14)NeuAc(2)	13,859.85518	13,851.83678	− 0.8
C_577_H_900_N_132_O_235_S_13_	HexNAc(8)Hex(9)NeuAc(2)	13,861.90851	13,853.89015	− 3.9
C_578_H_903_N_131_O_236_S_13_	HexNAc(8)Hex(10)NeuAc(1)Fuc(1)	13,878.92386	13,870.90547	− 0.5
C_580_H_906_N_132_O_237_S_13_	HexNAc(9)Hex(10)NeuAc(1)	13,935.94534	13,927.92292	− 4.1
C_580_H_904_N_132_O_238_S_13_	HexNAc(7)Hex(9)NeuAc(3)	13,949.92460	13,941.90620	− 0.6
C_581_H_907_N_131_O_240_S_13_	HexNAc(7)Hex(11)NeuAc(2)	13,982.93489	13,974.91643	− 1.4
C_583_H_910_N_132_O_240_S_13_	HexNAc(8)Hex(10)NeuAc(2)	14,023.96143	14,015.94298	+ 0.6
C_585_H_915_N_133_O_239_S_13_	HexNAc(11)Hex(10)NeuAc(0)	14,051.00873	14,042.99026	0.0
C_585_H_913_N_133_O_240_S_13_	HexNAc(9)Hex(9)NeuAc(2)	14,064.98799	14,056.96953	− 3.5
C_585_H_918_N_133_O_241_S_13_	HexNAc(7)Hex(8)NeuAc(4)	14,078.96726	14,070.94879	− 1.4
C_586_H_916_N_132_O_242_S_13_	HexNAc(9)Hex(11)NeuAc(1)	14,097.99826	14,089.97976	+ 0.1
C_586_H_914_N_132_O_243_S_13_	HexNAc(7)Hex(10)NeuAc(3)	14,112.97936	14,103.95902	+ 0.5
C_589_H_919_N_135_O_240_S_13_	HexNAc(11)Hex(7)NeuAc(2)	14,148.04112	14,139.02262	− 5.3
C_589_H_919_N_135_O_240_S_13_	HexNAc(7)Hex(11)NeuAc(3)	14,148.04112	14,139.02262	+ 0.2
C_588_H_917_N_133_O_243_S_13_	HexNAc(8)Hex(9)NeuAc(3)	14,154.00593	14,144.98557	− 0.6
C_589_H_920_N_132_O_244_S_13_	HexNAc(8)Hex(10)NeuAc(2)Fuc(1)	14,171.02128	14,162.00089	− 3.8
C_589_H_920_N_132_O_245_S_13_	HexNAc(8)Hex(11)NeuAc(2)	14,187.01620	14,177.99580	− 2.6
C_592_H_925_N_135_O_242_S_13_	HexNAc(12)Hex(8)NeuAc(1)	14,222.07979	14,213.05940	− 5.8
C_591_H_923_N_133_O_245_S_13_	HexNAc(9)Hex(10)NeuAc(2)	14,228.04276	14,219.02235	− 5.6
C_594_H_929_N_133_O_247_S_13_	HexNAc(10)Hex(11)NeuAc(1)	14,302.07960	14,293.05913	− 3.6
C_594_H_927_N_133_O_248_S_13_	HexNAc(8)Hex(10)NeuAc(3)	14,316.05886	14,307.03839	− 0.2
C_596_H_932_N_134_O_247_S_13_	HexNAc(11)Hex(10)NeuAc(1)	14,343.10616	14,334.08567	− 0.3
C_596_H_938_N_134_O_248_S_13_	HexNAc(9)Hex(9)NeuAc(3)	14,357.08543	14,348.06494	− 2.8
C_597_H_933_N_133_O_250_S_13_	HexNAc(9)Hex(11)NeuAc(2)	14,389.09569	14,381.07517	− 1.1
C_598_H_933_N_135_O_248_S_13_	HexNAc(10)Hex(8)NeuAc(3)	14,398.11199	14,389.09149	− 3.6
C_600_H_936_N_136_O_248_S_13_	HexNAc(11)Hex(7)NeuAc(3)	14,439.13856	14,430.11804	− 6.6
C_600_H_937_N_133_O_252_S_13_	HexNAc(8)Hex(10)NeuAc(3)Fuc(1)	14,462.11688	14,453.09630	− 4.0
C_600_H_939_N_133_O_252_S_13_	HexNAc(10)Hex(12)NeuAc(1)	14,464.13253	14,455.11195	− 2.7
C_600_H_937_N_133_O_253_S_13_	HexNAc(8)Hex(11)NeuAc(3)	14,478.11180	14,469.09122	+ 0.1
C_603_H_942_N_136_O_250_S_13_	HexNAc(12)Hex(8)NeuAc(2)	14,513.17539	14,504.15482	− 4.9
C_604_H_945_N_135_O_252_S_13_	HexNAc(12)Hex(10)NeuAc(1)	14,546.18566	14,537.16505	− 3.5
C_603_H_943_N_133_O_255_S_13_	HexNAc(9)Hex(12)NeuAc(2)	14,552.14862	14,543.12800	− 1.9
C_605_H_944_N_134_O_256_S_13_	HexNAc(8)Hex(10)NeuAc(4)	14,607.15445	14,598.13381	− 0.2
C_606_H_947_N_133_O_258_S_13_	HexNAc(8)Hex(12)NeuAc(3)	14,640.16472	14,631.14404	+ 1.1
C_607_H_947_N_135_O_256_S_13_	HexNAc(9)Hex(9)NeuAc(4)	14,648.18102	14,639.16036	− 2.0
C_608_H_950_N_134_O_258_S_13_	HexNAc(9)Hex(11)NeuAc(3)	14,681.19126	14,672.17059	+ 0.1
C_611_H_956_N_134_O_260_S_13_	HexNAc(10)Hex(12)NeuAc(2)	14,755.22812	14,746.20737	− 2.0
C_611_H_954_N_134_O_261_S_13_	HexNAc(8)Hex(11)NeuAc(4)	14,769.20738	14,760.18663	− 1.8
C_614_H_960_N_134_O_262_S_13_	HexNAc(9)Hex(11)NeuAc(3)Fuc(1)	14,827.24930	14,818.22850	− 3.2
C_614_H_962_N_134_O_262_S_13_	HexNAc(11)Hex(13)NeuAc(1)	14,829.26485	14,820.24415	− 1.6
C_616_H_961_N_135_O_264_S_13_	HexNAc(8)Hex(10)NeuAc(5)	14,898.25189	14,889.22923	− 4.3
C_619_H_967_N_135_O_268_S_13_	HexNAc(9)Hex(11)NeuAc(4)	14,972.28687	14,963.26601	− 0.5
C_621_H_970_N_136_O_266_S_13_	HexNAc(10)Hex(10)NeuAc(4)	15,013.31343	15,004.29255	− 2.3
C_622_H_973_N_135_O_268_S_13_	HexNAc(10)Hex(12)NeuAc(3)	15,046.32370	15,037.30279	− 2.0
C_623_H_967_N_137_O_269_S_13_	HexNAc(5)Hex(6)NeuAc(10)	15,096.27779	15,087.25690	− 0.6
C_630_H_984_N_136_O_274_S_13_	HexNAc(9)Hex(11)NeuAc(5)	15,263.38245	15,254.36142	− 0.5
C_632_H_987_N_137_O_274_S_13_	HexNAc(10)Hex(10)NeuAc(5)	15,304.40901	15,295.38797	− 2.9
C_633_H_990_N_136_O_276_S_13_	HexNAc(10)Hex(12)NeuAc(4)	15,337.41928	15,328.39820	− 1.8
C_644_H_1007_N_137_O_284_S_13_	HexNAc(10)Hex(12)NeuAc(5)	15,628.51485	15,619.49362	− 1.1

**Table 3:** Compilation of all the identified hCGβ glycoforms in the hCG sample analyzed by nano-LC-HRMS using DFA at 0.05% and in AIF mode (10 eV): elemental composition, proposed monosaccharide composition, theoretical maximum isotopic mass, theoretical monoisotopic mass, and the error in ppm

Elemental composition	Proposed monosaccharide composition	Theoretical maximum isotope mass	Theoretical monoisotopic mass	Error (ppm)
C_857_H_1387_N_211_O_336_S_13_	HexNAc(14)Hex(11)NeuAc(1)	20,438.45912	20,426.43017	− 7.9
C_856_H_1384_N_206_O_345_S_13_	HexNAc(6)Hex(16)NeuAc(4)	20,497.37413	20,485.34555	+ 4.7
C_859_H_1388_N_206_O_348_S_13_	HexNAc(5)Hex(16)NeuAc(5)	20,585.39077	20,573.36160	+ 4.0
C_878_H_1419_N_211_O_355_S_13_	HexNAc(11)Hex(13)NeuAc(4)	21,027.61537	21,014.58394	− 2.4
C_880_H_1427_N_209_O_358_S_13_	HexNAc(13)Hex(18)NeuAc(0)	21,079.65664	21,066.62514	− 4.7
C_882_H_1429_N_213_O_353_S_13_	HexNAc(17)Hex(13)NeuAc(0)	21,081.70996	21,068.67852	− 6.8
C_880_H_1420_N_212_O_356_S_13_	HexNAc(10)Hex(11)NeuAc(6)	21,082.62120	21,069.58976	− 2.0
C_880_H_1428_N_206_O_363_S_13_	HexNAc(10)Hex(22)NeuAc(0)	21,118.62988	21,105.59832	− 6.2
C_884_H_1428_N_214_O_355_S_13_	HexNAc(14)Hex(10)NeuAc(4)	21,150.69505	21,137.66360	− 7.2
C_881_H_1424_N_208_O_363_S_13_	HexNAc(7)Hex(17)NeuAc(5)	21,141.57317	21,154.60470	+ 2.9
C_889_H_1436_N_212_O_363_S_13_	HexNAc(11)Hex(13)NeuAc(5)	21,318.71093	21,305.67936	+ 0.6
C_888_H_1439_N_207_O_369_S_13_	HexNAc(9)Hex(21)NeuAc(2)	21,335.68861	21,322.65656	− 0.2
C_891_H_1444_N_210_O_366_S_13_	HexNAc(13)Hex(18)NeuAc(1)	21,370.75220	21,357.72056	− 4.3
C_894_H_1450_N_210_O_368_S_13_	HexNAc(14)Hex(19)NeuAc(0)	21,444.78902	21,431.75734	− 2.7
C_896_H_1452_N_214_O_363_S_13_	HexNAc(18)Hex(14)NeuAc(0)	21,446.84234	21,433.81071	− 2.5
C_895_H_1448_N_212_O_367_S_13_	HexNAc(13)Hex(15)NeuAc(3)	21,466.78458	21,453.75292	− 2.1
C_897_H_1452_N_210_O_372_S_13_	HexNAc(11)Hex(18)NeuAc(3)	21,546.78437	21,533.75265	− 5.5
C_900_H_1460_N_210_O_373_S_13_	HexNAc(14)Hex(20)NeuAc(0)	21,606.84193	21,593.81016	− 2.7
C_902_H_1452_N_214_O_373_S_13_	HexNAc(8)Hex(10)NeuAc(10)	21,678.79158	21,665.75986	− 2.0
C_905_H_1467_N_211_O_376_S_13_	HexNAc(14)Hex(19)NeuAc(1)	21,735.88458	21,722.85275	− 5.0
C_903_H_1458_N_210_O_379_S_13_	HexNAc(7)Hex(17)NeuAc(7)	21,736.79581	21,723.76400	+ 3.3
C_907_H_1469_N_215_O_371_S_13_	HexNAc(18)Hex(14)NeuAc(1)	21,737.93790	21,724.90613	− 5.4
C_904_H_1468_N_206_O_383_S_13_	HexNAc(10)Hex(26)NeuAc(0)	21,766.84152	21,753.80961	− 0.5
C_905_H_1461_N_211_O_379_S_13_	HexNAc(8)Hex(16)NeuAc(7)	21,777.82237	21,764.79055	− 1.7
C_907_H_1463_N_215_O_374_S_13_	HexNAc(12)Hex(11)NeuAc(7)	21,779.87569	21,766.84392	− 3.7
C_906_H_1464_N_210_O_381_S_13_	HexNAc(8)Hex(18)NeuAc(6)	21,797.80078	21,810.83263	− 0.6
C_909_H_1466_N_216_O_374_S_13_	HexNAc(13)Hex(10)NeuAc(7)	21,807.87047	21,820.90225	− 3.4
C_908_H_1464_N_214_O_377_S_13_	HexNAc(10)Hex(12)NeuAc(8)	21,813.83342	21,826.86310	− 0.9
C_911_H_1471_N_217_O_373_S_13_	HexNAc(16)Hex(10)NeuAc(5)	21,834.91775	21,847.94954	− 5.9
C_911_H_1477_N_211_O_381_S_13_	HexNAc(14)Hex(20)NeuAc(1)	21,884.90558	21,897.93748	− 2.7
C_913_H_1479_N_215_O_376_S_13_	HexNAc(18)Hex(15)NeuAc(1)	21,886.95895	21,899.99081	− 6.4
C_913_H_1477_N_215_O_377_S_13_	HexNAc(16)Hex(14)NeuAc(3)	21,900.93822	21,913.97007	− 5.8
C_913_H_1475_N_215_O_378_S_13_	HexNAc(14)Hex(13)NeuAc(5)	21,914.91748	21,927.94934	− 5.4
C_912_H_1471_N_213_O_382_S_13_	HexNAc(9)Hex(14)NeuAc(8)	21,934.85969	21,947.89157	− 1.8
C_914_H_1474_N_214_O_382_S_13_	HexNAc(10)Hex(13)NeuAc(8)	21,975.88624	21,988.91813	− 2.3
C_915_H_1481_N_213_O_382_S_13_	HexNAc(14)Hex(17)NeuAc(3)	21,980.93794	21,993.96986	− 3.4
C_915_H_1474_N_216_O_380_S_13_	HexNAc(11)Hex(10)NeuAc(9)	21,983.90256	21,996.93442	− 1.3
C_918_H_1490_N_210_O_384_S_13_	HexNAc(14)Hex(19)NeuAc(0)Fuc(4)	22,015.98897	22,029.02099	− 5.8
C_918_H_1486_N_216_O_379_S_13_	HexNAc(18)Hex(14)NeuAc(2)	22,016.00154	22,029.03345	− 6.2
C_916_H_1477_N_215_O_382_S_13_	HexNAc(11)Hex(12)NeuAc(8)	22,016.91279	22,029.94469	− 1.0
C_915_H_1475_N_213_O_385_S_13_	HexNAc(8)Hex(14)NeuAc(9)	22,022.87573	22,035.90766	+ 1.0
C_914_H_1485_N_205_O_393_S_13_	HexNAc(9)Hex(29)NeuAc(0)	22,036.88871	22,049.92077	− 4.6
C_915_H_1485_N_207_O_391_S_13_	HexNAc(10)Hex(26)NeuAc(1)	22,044.90503	22,057.93707	− 2.8
C_918_H_1487_N_213_O_384_S_13_	HexNAc(15)Hex(18)NeuAc(2)	22,054.97472	22,068.00669	− 9.0
C_916_H_1478_N_212_O_387_S_13_	HexNAc(8)Hex(16)NeuAc(8)	22,055.88596	22,068.91792	− 1.8
C_917_H_1483_N_211_O_388_S_13_	HexNAc(10)Hex(19)NeuAc(5)	22,074.91693	22,087.94892	− 2.5
C_917_H_1488_N_208_O_391_S_13_	HexNAc(11)Hex(25)NeuAc(1)	22,085.93158	22,098.96363	− 2.8
C_917_H_1481_N_211_O_389_S_13_	HexNAc(8)Hex(18)NeuAc(7)	22,088.89620	22,101.92818	+ 0.6
C_921_H_1490_N_216_O_382_S_13_	HexNAc(17)Hex(14)NeuAc(3)	22,104.01759	22,117.04954	− 4.3
C_918_H_1486_N_210_O_390_S_13_	HexNAc(10)Hex(21)NeuAc(4)	22,107.92716	22,120.95918	− 2.6
C_919_H_1487_N_209_O_393_S_13_	HexNAc(8)Hex(22)NeuAc(5)	22,154.91666	22,167.94871	− 1.2
C_922_H_1489_N_215_O_386_S_13_	HexNAc(13)Hex(14)NeuAc(6)	22,164.98635	22,178.01833	− 4.1
C_920_H_1492_N_208_O_394_S_13_	HexNAc(10)Hex(25)NeuAc(2)	22,173.94762	22,186.97971	− 0.5
C_920_H_1490_N_208_O_395_S_13_	HexNAc(8)Hex(24)NeuAc(4)	22,187.92689	22,200.95898	+ 0.8
C_924_H_1500_N_210_O_393_S_13_	HexNAc(14)Hex(24)NeuAc(0)	22,242.02145	22,255.05356	− 3.9
C_922_H_1496_N_206_O_399_S_13_	HexNAc(8)Hex(28)NeuAc(2)	22,253.94735	22,266.97950	+ 1.4
C_924_H_1496_N_210_O_395_S_13_	HexNAc(10)Hex(22)NeuAc(4)	22,269.97998	22,283.01209	− 1.9
C_924_H_1499_N_207_O_399_S_13_	HexNAc(9)Hex(27)NeuAc(2)	22,294.97390	22,308.00606	− 1.5
C_929_H_1503_N_217_O_387_S_13_	HexNAc(18)Hex(14)NeuAc(3)	22,307.09696	22,320.12700	− 7.4
C_929_H_1507_N_211_O_393_S_13_	HexNAc(14)Hex(20)NeuAc(1)Fuc(3)	22,323.07930	22,336.11146	− 5.7
C_928_H_1506_N_212_O_393_S_13_	HexNAc(16)Hex(22)NeuAc(0)	22,324.07455	22,337.10668	− 5.2
C_926_H_1497_N_211_O_396_S_13_	HexNAc(9)Hex(20)NeuAc(6)	22,324.98580	22,338.01791	− 3.8
C_929_H_1504_N_214_O_392_S_13_	HexNAc(15)Hex(18)NeuAc(3)	22,346.07014	22,359.10223	− 5.7
C_930_H_1504_N_216_O_390_S_13_	HexNAc(16)Hex(15)NeuAc(4)	22,354.08646	22,367.11853	− 4.5
C_930_H_1509_N_213_O_393_S_13_	HexNAc(17)Hex(21)NeuAc(0)	22,365.10110	22,378.13323	− 5.4
C_928_H_1498_N_212_O_397_S_13_	HexNAc(8)Hex(18)NeuAc(8)	22,379.99161	22,393.02373	− 0.1
C_932_H_1509_N_217_O_389_S_13_	HexNAc(19)Hex(15)NeuAc(2)	22,381.13374	22,394.16582	− 7.1
C_931_H_1507_N_215_O_392_S_13_	HexNAc(16)Hex(17)NeuAc(3)	22,387.09669	22,400.12879	− 5.7
C_930_H_1498_N_216_O_393_S_13_	HexNAc(10)Hex(12)NeuAc(10)	22,396.02425	22,409.05632	− 0.7
C_934_H_1512_N_218_O_389_S_13_	HexNAc(20)Hex(14)NeuAc(2)	22,422.16029	22,435.19238	− 5.8
C_931_H_1509_N_209_O_402_S_13_	HexNAc(10)Hex(25)NeuAc(3)	22,465.04304	22,478.07526	− 2.0
C_937_H_1513_N_221_O_388_S_13_	HexNAc(20)Hex(9)NeuAc(5)	22,485.18242	22,498.21449	− 7.5
C_934_H_1512_N_212_O_400_S_13_	HexNAc(12)Hex(21)NeuAc(4)	22,514.08591	22,527.11811	− 2.0
C_933_H_1515_N_207_O_406_S_13_	HexNAc(10)Hex(29)NeuAc(1)	22,531.06350	22,544.09578	− 2.8
C_936_H_1517_N_213_O_399_S_13_	HexNAc(15)Hex(21)NeuAc(2)	22,541.13319	22,554.16540	− 6.7
C_938_H_1512_N_220_O_392_S_13_	HexNAc(16)Hex(9)NeuAc(8)	22,546.15118	22,559.18323	− 4.4
C_938_H_1514_N_214_O_402_S_13_	HexNAc(10)Hex(17)NeuAc(8)	22,624.09753	22,637.12759	0.0
C_941_H_1516_N_220_O_395_S_13_	HexNAc(15)Hex(9)NeuAc(9)	22,634.16723	22,647.19937	− 3.9
C_939_H_1524_N_210_O_406_S_13_	HexNAc(13)Hex(26)NeuAc(1)	22,654.14315	22,667.17545	− 3.7
C_939_H_1515_N_213_O_405_S_13_	HexNAc(8)Hex(18)NeuAc(9)	22,671.08703	22,684.11928	− 0.1
C_943_H_1526_N_218_O_397_S_13_	HexNAc(19)Hex(15)NeuAc(3)	22,672.22916	22,685.26136	− 5.8
C_938_H_1520_N_208_O_410_S_13_	HexNAc(8)Hex(27)NeuAc(4)	22,674.08536	22,687.11769	+ 1.4
C_940_H_1520_N_212_O_406_S_13_	HexNAc(10)Hex(21)NeuAc(6)	22,690.11799	22,703.15027	+ 0.3
C_944_H_1524_N_220_O_396_S_13_	HexNAc(18)Hex(11)NeuAc(6)	22,694.22474	22,707.25692	− 5.4
C_943_H_1525_N_215_O_403_S_13_	HexNAc(14)Hex(18)NeuAc(5)	22,725.18160	22,783.21386	− 1.1
C_942_H_1521_N_213_O_407_S_13_	HexNAc(9)Hex(19)NeuAc(8)	22,745.12381	22,758.15610	− 1.0
C_942_H_1526_N_210_O_410_S_13_	HexNAc(10)Hex(25)NeuAc(4)	22,756.13845	22,769.17080	− 0.5
C_943_H_1526_N_212_O_408_S_13_	HexNAc(11)Hex(22)NeuAc(5)	22,764.15477	22,777.18709	− 4.1
C_946_H_1533_N_215_O_404_S_13_	HexNAc(17)Hex(20)NeuAc(2)	22,785.23911	22,798.27142	− 4.9
C_945_H_1524_N_216_O_405_S_13_	HexNAc(11)Hex(15)NeuAc(9)	22,794.16668	22,807.19895	− 3.4
C_948_H_1531_N_219_O_401_S_13_	HexNAc(17)Hex(13)NeuAc(6)	22,815.25101	22,828.28327	− 6.6
C_944_H_1530_N_208_O_415_S_13_	HexNAc(8)Hex(28)NeuAc(4)	22,836.13818	22,849.17059	− 0.1
C_945_H_1530_N_210_O_413_S_13_	HexNAc(9)Hex(25)NeuAc(5)	22,844.15450	22,857.18688	− 1.7
C_948_H_1532_N_216_O_406_S_13_	HexNAc(14)Hex(17)NeuAc(6)	22,854.22420	22,868.25864	− 2.5
C_950_H_1533_N_217_O_407_S_13_	HexNAc(13)Hex(15)NeuAc(8)	22,909.23000	22,923.26447	+ 0.8
C_947_H_1536_N_208_O_417_S_13_	HexNAc(9)Hex(29)NeuAc(3)	22,910.17496	22,924.20956	+ 0.6
C_949_H_1536_N_212_O_413_S_13_	HexNAc(11)Hex(23)NeuAc(5)	22,926.20760	22,940.24214	− 0.1
C_950_H_1541_N_211_O_414_S_13_	HexNAc(13)Hex(26)NeuAc(2)	22,945.23856	22,959.27314	− 5.4
C_951_H_1535_N_213_O_413_S_13_	HexNAc(8)Hex(18)NeuAc(9)Fuc(2)	22,963.20285	22,977.23740	− 0.5
C_951_H_1537_N_213_O_414_S_13_	HexNAc(10)Hex(21)NeuAc(7)	22,981.21341	22,995.24797	+ 0.1
C_953_H_1539_N_217_O_409_S_13_	HexNAc(14)Hex(16)NeuAc(7)	22,983.26678	22,997.30129	− 2.7
C_950_H_1540_N_208_O_420_S_13_	HexNAc(8)Hex(29)NeuAc(2)	22,998.19100	23,012.22565	− 1.7
C_953_H_1538_N_214_O_415_S_13_	HexNAc(9)Hex(19)NeuAc(9)	23,036.21922	23,050.25379	− 1.1
C_954_H_1543_N_213_O_416_S_13_	HexNAc(11)Hex(22)NeuAc(6)	23,055.25019	23,069.28479	0.0
C_956_H_1545_N_217_O_411_S_13_	HexNAc(15)Hex(17)NeuAc(6)	23,057.30356	23,071.33811	− 2.3
C_958_H_1545_N_221_O_407_S_13_	HexNAc(17)Hex(11)NeuAc(8)	23,073.33620	23,087.37070	− 3.5
C_956_H_1541_N_217_O_413_S_13_	HexNAc(11)Hex(15)NeuAc(10)	23,085.26209	23,099.29664	− 0.1
C_954_H_1544_N_210_O_421_S_13_	HexNAc(8)Hex(26)NeuAc(6)	23,094.22337	23,108.25023	− 0.9
C_957_H_1549_N_213_O_418_S_13_	HexNAc(12)Hex(23)NeuAc(5)	23,129.28697	23,143.32161	− 1.0
C_957_H_1547_N_213_O_419_S_13_	HexNAc(10)Hex(22)NeuAc(7)	23,143.26623	23,157.30087	0.0
C_963_H_1552_N_222_O_410_S_13_	HexNAc(17)Hex(10)NeuAc(9)	23,202.37879	23,216.41334	− 2.2
C_960_H_1555_N_213_O_420_S_13_	HexNAc(13)Hex(24)NeuAc(4)	23,203.32375	23,217.35843	+ 0.1
C_960_H_1553_N_213_O_421_S_13_	HexNAc(11)Hex(23)NeuAc(6)	23,217.30301	23,231.33770	+ 1.8
C_962_H_1555_N_217_O_416_S_13_	HexNAc(15)Hex(18)NeuAc(6)	23,219.35639	23,233.39102	− 1.2
C_963_H_1555_N_213_O_421_S_13_	HexNAc(8)Hex(18)NeuAc(9)Fuc(4)	23,255.31866	23,269.35338	− 1.3
C_964_H_1563_N_215_O_419_S_13_	HexNAc(17)Hex(23)NeuAc(2)	23,271.39758	23,285.43228	− 2.3
C_962_H_1554_N_214_O_422_S_13_	HexNAc(10)Hex(21)NeuAc(8)	23,272.30883	23,286.34352	+ 0.3
C_964_H_1556_N_218_O_417_S_13_	HexNAc(14)Hex(16)NeuAc(8)	23,274.36220	23,288.39684	0.0
C_964_H_1561_N_215_O_420_S_13_	HexNAc(15)Hex(22)NeuAc(4)	23,285.37685	23,299.41155	+ 0.1
C_963_H_1559_N_213_O_423_S_13_	HexNAc(12)Hex(24)NeuAc(5)	23,291.33979	23,305.37452	− 2.0
C_963_H_1552_N_216_O_421_S_13_	HexNAc(9)Hex(17)NeuAc(11)	23,294.30441	23,308.33907	+ 2.1
C_965_H_1560_N_214_O_424_S_13_	HexNAc(11)Hex(22)NeuAc(7)	23,346.34561	23,360.38034	− 0.2
C_964_H_1558_N_212_O_427_S_13_	HexNAc(8)Hex(24)NeuAc(8)	23,352.30855	23,366.34331	− 1.2
C_965_H_1561_N_211_O_429_S_13_	HexNAc(8)Hex(26)NeuAc(7)	23,385.31878	23,399.35357	− 1.2
C_967_H_1563_N_215_O_424_S_13_	HexNAc(12)Hex(21)NeuAc(7)	23,387.37216	23,401.40689	+ 0.0
C_967_H_1559_N_215_O_426_S_13_	HexNAc(8)Hex(19)NeuAc(11)	23,415.33068	23,429.36542	+ 2.6
C_969_H_1567_N_213_O_426_S_13_	HexNAc(10)Hex(21)NeuAc(7)Fuc(3)	23,419.89714	23,433.42194	− 0.2
C_968_H_1566_N_214_O_426_S_13_	HexNAc(12)Hex(23)NeuAc(6)	23,420.38239	23,434.41716	− 1.8
C_969_H_1564_N_216_O_425_S_13_	HexNAc(11)Hex(19)NeuAc(9)	23,442.37797	23,456.41272	− 1.1
C_971_H_1566_N_220_O_420_S_13_	HexNAc(15)Hex(14)NeuAc(9)	23,444.43134	23,458.46604	− 3.5
C_971_H_1571_N_217_O_423_S_13_	HexNAc(16)Hex(20)NeuAc(5)	23,455.44599	23,469.48074	− 0.2
C_969_H_1570_N_210_O_433_S_13_	HexNAc(9)Hex(29)NeuAc(5)	23,492.36579	23,506.40066	+ 1.3
C_971_H_1570_N_214_O_428_S_13_	HexNAc(11)Hex(22)NeuAc(7)Fuc(1)	23,492.40352	23,506.43833	− 0.1
C_972_H_1573_N_213_O_429_S_13_	HexNAc(11)Hex(23)NeuAc(6)Fuc(2)	23,509.41883	23,523.45368	+ 1.0
C_973_H_1570_N_218_O_425_S_13_	HexNAc(13)Hex(17)NeuAc(9)	23,538.46583	23,524.43107	− 0.5
C_974_H_1574_N_214_O_430_S_13_	HexNAc(10)Hex(21)NeuAc(8)Fuc(2)	23,564.42464	23,578.45950	− 1.3
C_976_H_1576_N_218_O_425_S_13_	HexNAc(14)Hex(16)NeuAc(8)Fuc(2)	23,566.47802	23,580.51282	+ 0.2
C_976_H_1578_N_218_O_426_S_13_	HexNAc(16)Hex(19)NeuAc(6)	23,584.48858	23,598.52339	− 1.9
C_975_H_1574_N_216_O_430_S_13_	HexNAc(11)Hex(20)NeuAc(9)	23,604.43079	23,618.46562	+ 0.7
C_975_H_1579_N_213_O_433_S_13_	HexNAc(12)Hex(26)NeuAc(5)	23,615.44544	23,629.48033	+ 1.3
C_976_H_1577_N_215_O_432_S_13_	HexNAc(11)Hex(22)NeuAc(8)	23,637.44102	23,651.47588	0.0
C_978_H_1583_N_213_O_433_S_13_	HexNAc(11)Hex(23)NeuAc(6)Fuc(3)	23,655.47674	23,669.51167	− 2.4
C_977_H_1582_N_214_O_433_S_13_	HexNAc(13)Hex(25)NeuAc(5)	23,656.47199	23,670.50688	+ 0.1
C_978_H_1580_N_216_O_432_S_13_	HexNAc(12)Hex(21)NeuAc(8)	23,678.46757	23,692.50244	+ 0.7
C_980_H_1585_N_217_O_431_S_13_	HexNAc(15)Hex(21)NeuAc(6)	23,705.51486	23,719.54973	+ 0.5
C_980_H_1584_N_214_O_434_S_13_	HexNAc(10)Hex(21)NeuAc(8)Fuc(3)	23,710.48255	23,724.51749	− 0.7
C_979_H_1583_N_215_O_434_S_13_	HexNAc(12)Hex(23)NeuAc(7)	23,711.47780	23,725.51270	− 0.7
C_979_H_1581_N_215_O_435_S_13_	HexNAc(10)Hex(22)NeuAc(9)	23,725.45707	23,739.49197	+ 2.6
C_982_H_1583_N_221_O_428_S_13_	HexNAc(15)Hex(14)NeuAc(10)	23,735.52676	23,749.56158	− 0.1
C_980_H_1586_N_214_O_436_S_13_	HexNAc(12)Hex(25)NeuAc(6)	23,744.48803	23,758.52297	+ 0.6
C_984_H_1586_N_222_O_428_S_13_	HexNAc(16)Hex(13)NeuAc(10)	23,776.55331	23,790.58814	+ 1.6
C_983_H_1584_N_220_O_431_S_13_	HexNAc(13)Hex(15)NeuAc(11)	23,782.51625	23,796.55111	− 0.4
C_982_H_1587_N_215_O_436_S_13_	HexNAc(11)Hex(22)NeuAc(8)Fuc(1)	23,783.49893	23,797.53387	0.1
C_985_H_1586_N_224_O_426_S_13_	HexNAc(17)Hex(10)NeuAc(11)	23,784.56963	23,798.60443	− 4.0
C_982_H_1587_N_215_O_437_S_13_	HexNAc(11)Hex(23)NeuAc(8)	23,799.49385	23,813.52879	+ 0.9
C_984_H_1593_N_213_O_437_S_13_	HexNAc(11)Hex(23)NeuAc(6)Fuc(4)	23,801.53465	23,815.56965	− 0.7
C_985_H_1592_N_218_O_434_S_13_	HexNAc(15)Hex(20)NeuAc(7)	23,834.55745	23,848.59237	− 1.2
C_984_H_1588_N_216_O_438_S_13_	HexNAc(10)Hex(21)NeuAc(10)	23,854.49966	23,868.53461	+ 1.1
C_986_H_1590_N_220_O_433_S_13_	HexNAc(14)Hex(16)NeuAc(10)	23,856.55303	23,870.58793	+ 1.1
C_984_H_1593_N_213_O_441_S_13_	HexNAc(11)Hex(27)NeuAc(6)	23,865.51431	23,879.54931	+ 2.0
C_987_H_1595_N_219_O_434_S_13_	HexNAc(16)Hex(19)NeuAc(7)	23,875.58400	23,889.61893	− 1.2
C_984_H_1591_N_213_O_442_S_13_	HexNAc(9)Hex(26)NeuAc(8)	23,879.49357	23,893.52858	− 0.5
C_986_H_1591_N_217_O_438_S_13_	HexNAc(11)Hex(20)NeuAc(10)	23,895.52621	23,909.56116	+ 1.3
C_987_H_1594_N_216_O_440_S_13_	HexNAc(11)Hex(22)NeuAc(9)	23,928.53644	23,942.57143	+ 0.1
C_990_H_1603_N_213_O_441_S_13_	HexNAc(11)Hex(23)NeuAc(6)Fuc(5)	23,947.59256	23,961.62764	+ 0.1
C_991_H_1602_N_218_O_439_S_13_	HexNAc(15)Hex(21)NeuAc(7)	23,996.61027	24,010.64528	− 3.3
C_992_H_1604_N_214_O_442_S_13_	HexNAc(10)Hex(21)NeuAc(8)Fuc(5)	24,002.59837	24,016.63346	− 2.4
C_991_H_1607_N_215_O_442_S_13_	HexNAc(16)Hex(27)NeuAc(3)	24,007.62492	24,021.65998	− 3.7
C_993_H_1602_N_222_O_435_S_13_	HexNAc(17)Hex(15)NeuAc(9)	24,012.64291	24,026.67786	− 1.2
C_992_H_1598_N_220_O_439_S_13_	HexNAc(12)Hex(16)NeuAc(12)	24,032.58512	24,046.62010	+ 1.2
C_991_H_1603_N_215_O_444_S_13_	HexNAc(12)Hex(25)NeuAc(7)	24,035.58345	24,049.61851	+ 2.5
C_990_H_1601_N_213_O_447_S_13_	HexNAc(9)Hex(27)NeuAc(8)	24,041.54340	24,055.58148	+ 1.0
C_994_H_1605_N_221_O_437_S_13_	HexNAc(17)Hex(17)NeuAc(8)	24,045.65314	24,059.68813	− 1.2
C_992_H_1608_N_214_O_445_S_13_	HexNAc(14)Hex(28)NeuAc(4)	24,054.61442	24,068.64951	− 1.6
C_991_H_1604_N_212_O_449_S_13_	HexNAc(9)Hex(29)NeuAc(7)	24,074.55663	24,088.59174	+ 0.7
C_993_H_1604_N_216_O_444_S_13_	HexNAc(11)Hex(22)NeuAc(9)Fuc(1)	24,074.59435	24,088.62941	− 0.4
C_996_H_1603_N_225_O_434_S_13_	HexNAc(17)Hex(10)NeuAc(12)	24,075.66504	24,089.69998	− 3.6
C_997_H_1613_N_221_O_438_S_13_	HexNAc(20)Hex(19)NeuAc(5)	24,105.71066	24,119.74568	− 3.0
C_993_H_1607_N_213_O_449_S_13_	HexNAc(10)Hex(28)NeuAc(7)	24,115.58317	24,129.61830	+ 2.4
C_996_H_1610_N_216_O_447_S_13_	HexNAc(12)Hex(24)NeuAc(8)	24,164.62604	24,178.66115	+ 0.3
C_999_H_1617_N_219_O_443_S_13_	HexNAc(18)Hex(22)NeuAc(5)	24,185.71038	24,199.74547	− 0.9
C_1001_H_1610_N_226_O_437_S_13_	HexNAc(17)Hex(9)NeuAc(13)	24,204.70764	24,218.74261	− 2.5
C_998_H_1611_N_217_O_448_S_13_	HexNAc(11)Hex(22)NeuAc(10)	24,219.63186	24,233.66697	− 0.8
C_1000_H_1613_N_221_O_443_S_13_	HexNAc(15)Hex(17)NeuAc(10)	24,221.68523	24,235.72030	− 2.7
C_998_H_1614_N_214_O_452_S_13_	HexNAc(10)Hex(27)NeuAc(8)	24,244.62577	24,258.66094	0.0
C_1004_H_1618_N_226_O_438_S_13_	HexNAc(20)Hex(11)NeuAc(10)	24,264.76515	24,278.80017	− 5.5
C_1001_H_1621_N_217_O_448_S_13_	HexNAc(16)Hex(25)NeuAc(5)	24,265.71012	24,279.74526	− 4.2
C_1001_H_1617_N_217_O_450_S_13_	HexNAc(12)Hex(23)NeuAc(9)	24,293.66864	24,307.70379	− 0.8
C_1002_H_1621_N_213_O_457_S_13_	HexNAc(9)Hex(29)NeuAc(8)	24,365.65204	24,379.68728	− 0.2
C_1004_H_1623_N_217_O_452_S_13_	HexNAc(13)Hex(24)NeuAc(8)	24,367.70541	24,381.74060	− 0.9
C_1004_H_1621_N_217_O_453_S_13_	HexNAc(11)Hex(23)NeuAc(10)	24,381.68468	24,395.71987	− 1.6
C_1008_H_1633_N_213_O_453_S_13_	HexNAc(11)Hex(23)NeuAc(6)Fuc(8)	24,385.76628	24,399.80160	− 1.6
C_1008_H_1624_N_222_O_449_S_13_	HexNAc(14)Hex(16)NeuAc(12)	24,438.74387	24,452.77901	− 1.3
C_1013_H_1632_N_226_O_446_S_13_	HexNAc(19)Hex(12)NeuAc(11)	24,514.83402	24,528.86915	− 4.3
C_1015_H_1642_N_218_O_455_S_13_	HexNAc(15)Hex(21)NeuAc(7)Fuc(4)	24,580.84191	24,594.87722	− 3.1
C_1012_H_1634_N_218_O_458_S_13_	HexNAc(12)Hex(23)NeuAc(10)	24,584.76405	24,598.79932	+ 0.2
C_1014_H_1636_N_222_O_453_S_13_	HexNAc(16)Hex(18)NeuAc(10)	24,586.81742	24,600.85265	− 1.9
C_1015_H_1641_N_221_O_454_S_13_	HexNAc(18)Hex(21)NeuAc(7)	24,605.84839	24,619.88364	− 3.8
C_1021_H_1641_N_227_O_453_S_13_	HexNAc(16)Hex(10)NeuAc(15)	24,745.87192	24,760.90932	− 2.3
C_1030_H_1666_N_218_O_472_S_13_	HexNAc(14)Hex(27)NeuAc(8)	25,056.94326	25,071.98096	− 2.3

## Conclusion and perspectives

In this study, the nano-LC-HRMS analytical method previously developed was further optimized in order to find the best compromise between the separation of the hCG glycoforms, the non-detection of adduct forms, and an increase in sensitivity leading to a higher number of glycoforms detected. The optimization was carried out along several parameters, the nano-LC ion-pairing agent nature and content, and dissociation methods inherent of the mass spectrometer. As illustrated in the study, the ion-pairing agent has a strong influence on the separation of the species but also on their ionization and adduct formation, whereas the dissociation method of the mass spectrometer mainly influences on the adduct form dissociation and detection. Using DFA at 0.05% coupled with AIF mode at 10 eV drastically reduced the detection of adduct form and enhanced the MS peak intensities. The optimized method allowed the detection of a significantly higher number of hCG glycoforms, by a factor of 2 for hCGα and 6 for hCGβ. A better characterization of a sample containing hCG should facilitate the potential identification of some hCG glycoforms which could be biomarkers of pregnancy pathologies. The application of the optimized nano-LC-HRMS method to other glycoproteins and real biological samples constitutes therefore the main perspective of this study.

## Data Availability

Data are available from the authors by request.
